# Dopamine in Health and Disease

**DOI:** 10.3390/biomedicines9111644

**Published:** 2021-11-09

**Authors:** Marc Ekker

**Affiliations:** Department of Biology, University of Ottawa, 30 Marie Curie, Ottawa, ON K1N 6N5, Canada; mekker4409@hotmail.com

The neurotransmitter dopamine (DA) is generally associated with Parkinson’s disease (PD). However, it is clear that DA exerts multiple functions within and outside the central nervous system (CNS). This Special Issue comprises eight review articles and five articles reporting original research. These highlight the roles of DA circuits within and outside the CNS, involvement of DA in diseases other than PD, as well as methodological development that allows for rapid advances in our understanding of DA function.

## 1. Review Articles

Two of the review articles in this Special Issue addressed the relationship between dopaminergic systems and conditions other than PD, notably, cancer and hypertension.

Thus, Ejma and colleagues [1] reviewed the relationship between PD and cancer. One of the factors associated with PD, aggregation of alpha-synuclein, has been shown to be involved in some types of neoplastic transformations, although its expression was found to be decreased in other types of cancer. The same applies to many of the other factors such as PINK1, PARKIN, DJ-1.

Qaddumi and Jose [2] examined the relationship between the dopaminergic system in the kidney, oxidative stress and the regulation of blood pressure. In the kidney, oxidative stress disrupts sodium homeostasis and causes hypertension through vasoconstriction. The relationship with sodium homeostasis is complex, and reactive oxygen species can increase or decrease renal sodium transport. Dopamine is produced in cells of the proximal tubules of the kidney and acts on receptors present on nephron segments. The authors reviewed the link between the various classes of DA receptors in the kidney, oxidative stress, and hypertension. Evidence suggests that the five DA receptor subtypes, their interactions amongst themselves and with other factors control ion transport in the renal tubules and reactive oxygen species production. These relationships may be different in kidney cells compared to other cell types.

The Special Issue also comprised two review articles that surveyed methodological developments.

Single-cell RNA sequencing has led to major advances in our understanding of a large number of biological processes and of pathologies. This is especially true in the neurosciences. Ma and Lim [3] have reviewed how single-cell and single-nucleus RNA sequencing have led to the successful characterization neuronal and glial cell populations associated with DA function and associated pathologies. Samples for such studies can originate from post-mortem substantia tissue, induced pluripotent or embryonic stem cells or animal models, and the advantages and challenges associated with each of these experimental systems are covered. The authors also address emerging tools and computational approaches to understand the transcriptome of DA neurons and other cell types associated with pathologies.

Michael R. Kilbourn [4] reviewed how positron emission tomography (PET) is used in vivo to image dopaminergic function in the central nervous system. Aspects of DA function that are amenable to PET include dopamine synthesis, vesicular storage, synaptic release, receptor binding and DA transport and reuptake. A survey of radiotracers is presented.

Additional review articles within this Special Issue examined some the roles of DA outside the CNS, its interaction with acetylcholine in the striatum, and how DA circuits in distinct brain regions associate with different pathological conditions. Finally, a final review examined DA’s role in how we synchronize our movements with that of others.

Franco and colleagues [5] reviewed roles for dopamine outside the CNS, with a particular emphasis on the gastrointestinal tract and the immune system. As the authors point out, DA is associated with more diseases than PD, in locations beyond the substantia nigra and striatum, something that is also addressed in other reviews of this Special Issue.

Jaromir Myslivecek [6] reviewed recent studies on the interaction between DA and acetylcholine in the striatum and how such interactions contribute to the regulation of locomotor activity and reward behavior. The role played by the circadian system in modulation the function of the two neurotransmitters is given particular attention. The author concludes by proposing a hierarchical model of increasing complexity for the interactions between DA and acetylcholine in the striatum.

Chen and colleagues [7] surveyed DA’s role in diverse brain regions and examined the hypothesis that DA’s behavioral function is linked to disease deficits in a neural circuit-dependent manner. Recent work surveyed by the authors suggests that distinct DA pathways are dysregulated in different diseases. The importance of new technologies, such as DA sensors and in vivo imaging, to examine DA function at the circuit level is outlined.

Gvirts Probolovski and Dahan [8] provided a synthesis of recent findings showing the pivotal role played by the dopaminergic system in our ability to synchronize our movements with those of others: interpersonal synchrony. Such alignment of behaviors in time is seen in multiple contexts including the mother–infant relationship, walking side-by-side or during conversation. Disorders that affect the dopaminergic system, such as attention deficit hyperactivity disorder (ADHD), are particularly examined. In short, whether motor and social skills are linked and share common neural mechanisms are at the center of this review.

## 2. Original Research

Two of the articles in this Special Issue reporting original research addressed DA transport.

Illiano and colleagues [9] examined the prefrontal cortex of rats lacking function of the dopamine transporter (DAT) during pre-adolescence. They observed changes in neuronal and glial homeostasis. More specifically, they saw a hyperactive phenotype accompanied by changes in glutamatergic neurotransmission, signs of neurodegeneration and glial activation.

In another original study, Cerantola and colleagues [10] examined the enteric nervous system of mice with genetic reductions in DAT. Thus, mice heterozygous for a DAT null mutation were examined for impact on enteric nervous system integrity. Muscle-myenteric plexus preparations were examined in contractility studies and gene/protein expression. The authors saw increased DA-mediated effects and reduced cholinergic response as a result of DAT reductions in the heterozygous DAT mice. Sustained tachykinergic and glutamatergic neurotransmission were also observed.

In another original research article dealing with transport, Kawahata and colleagues [11] used cultured mesencephalic neurons from wildtype and mutant mice to look at uptake of α-synuclein monomers and uptake. They observed that neurons derived from mice lacking the function of the D2 long type DA receptor (D_2L_) failed to uptake α-synuclein monomers. They conclude that D_2L_, coupled to fatty acid-binding protein 3 (FABP3) is critical for α-synuclein uptake by DA neurons which could be an important factor in the development of synucleinopathies, including PD.

Merhi and colleagues [12] used zebrafish to examine the function of *parla*, one of the two paralogs of the presenilin-associated rhomboid protease (*parl*) in this species. The human PARL gene had been associated with cases of PD [13]. Using CRISPR-mediated deletion, they showed that mutants lacking *parla* function showed reductions in the number of DA neurons, particularly in the olfactory bulb, and that this translated into impaired olfaction and locomotor behavior.

Finally, Mesman and colleagues [14], using targeted mutant mice, reported that *Tcf4*, a member of the E box family of transcriptional regulators, is involved in the specification of distinct subsets of mesodiencephalic DA neurons.

It is clear that DA is linked with more than PD and the nigrostriatal pathway. A number of creative research approaches have made the DA-related field of investigations one of the most vibrant and rapidly evolving. It is the editor’s wish that this Special Issue contributes to highlighting some of the field’s new trends.
